# ADVERSE EVENTS RELATED TO MECHANICAL VENTILATION IN A PEDIATRIC INTENSIVE CARE UNIT

**DOI:** 10.1590/1984-0462/2021/39/2019180

**Published:** 2020-08-26

**Authors:** Lana dos Santos Martins, Alexandre Rodrigues Ferreira, Fabiana Maria Kakehasi

**Affiliations:** a Pediatric Intensive Care Unit, Hospital das Clínicas, Universidade Federal de Minas Gerais, Belo Horizonte, MG, Brazil.; b School of Medicine, Universidade Federal de Minas Gerais, Belo Horizonte, MG, Brazil.

**Keywords:** Quality of health care, Respiration, artificial, Child, Adolescent, Intensive care unit, Adverse event, Qualidade dos cuidados de saúde, Ventilação mecânica, Crianças, Adolescentes, Unidades de terapia intensiva, Evento adverso

## Abstract

**Objective:**

To identify the prevalence and factors associated with adverse events (AE) related to invasive mechanical ventilation in patients admitted to the Pediatric Intensive Care Unit (PICU) of a tertiary public hospital.

**Methods:**

This is a cross-sectional study from July 2016 to June 2018, with data collected throughout patients’ routine care in the unit by the care team. Demographic, clinical and ventilatory characteristics and adverse events were analysed. The logistic regression model was used for multivariate analysis regarding the factors associated with AE.

**Results:**

Three hundred and six patients were included, with a total ventilation time of 2,155 days. Adverse events occurred in 66 patients (21.6%), and in 11 of those (16.7%) two AE occurred, totalling 77 events (36 AE per 1000 days of ventilation). The most common AE was post-extubation stridor (25.9%), followed by unplanned extubation (16.9%). Episodes occurred predominantly in the afternoon shift (49.3%) and associated with mild damage (54.6%). Multivariate analysis showed a higher occurrence of AE associated with length of stay of 7 days or more (*Odds Ratio* [OR]=2.6; 95% confidence interval [95%CI] 1.49-4.66; p=0.001).

**Conclusions:**

The results of the present study show a significant number of preventable adverse events, especially stridor after extubation and accidental extubation. The higher frequency of these events is associated with longer hospitalization.

## RESUMO

**Objetivo:**

Identificar a prevalência e os fatores associados a eventos adversos (EA) relacionados à ventilação mecânica (VM) invasiva em pacientes internados na Unidade de Terapia Intensiva Pediátrica (UTIP) de hospital público terciário.

**Métodos:**

Trata-se de estudo transversal realizado entre julho de 2016 e junho de 2018, com dados coletados ao longo da rotina de atendimento dos pacientes na unidade pela equipe assistencial. Neste estudo, foram analisados características demográficas, clínicas, ventilatórias e os EA ocorridos. O modelo de regressão logística foi utilizado para análise multivariada quanto aos fatores associados aos EA.

**Resultados:**

Neste estudo, foram incluídos 306 pacientes, com tempo de ventilação total de 2.155 dias. Ocorreram EA em 66 pacientes (21,6%), dos quais 11 (16,7%) sofreram dois EA, totalizando 77 eventos (36 EA por mil dias de ventilação). O EA mais comum foi o estridor pós-extubação (25,9%), seguido da extubação não planejada (16,9%). Os episódios ocorreram predominantemente no turno da tarde (49,3%) e associados a grau de dano leve (54,6%). Na análise multivariada, observou-se maior ocorrência de EA associado a tempo de internação igual ou superior a sete dias (*Odds Ratio* [OR]=2,6, intervalo de confiança de 95% [IC95%)]1,49-4,66, p=0,001).

**Conclusões:**

Evidenciou-se número significativo de EA que podem ser prevenidos, destacando-se o estridor pós-extubação e a extubação acidental, com ocorrência mais frequentemente associada ao maior tempo de internação.

## INTRODUCTION

The mechanical ventilation (MV) process for critically ill patients is complex, invasive and full of interactions. It encompasses a series of phases in which dynamism and interventionism in the care process are extremely important. This, in addition to the frequent severity of a patient’s condition, can produce a multitude of incidents that place the patient’s safety in potential or real danger to suffer from damage, and which can trigger serious sequelae and even death.^1^

An incident that results in damage is considered an adverse event (AE). It is usually unintentional because of care taking and not because of the natural evolution of the underlying disease.^2^ There are several AEs related to the use of invasive MV, such as: atelectasis, accidental extubation, selective intubation, ventilator-associated pneumonia (VAP), injury at the site of the orotracheal tube (OTT) fixation, trauma by aspiration, and obstruction of OTT through secretion. Some of these events are subject to the identification and/or direct intervention of the nurse, doctor or physiotherapist, and therefore are related to the quality of care.^3^ The occurrence of AE in patients in pediatric intensive care is common, ranging from 27 to 97 AE/thousand patients per day,^4^ ^,^ ^5^ and is especially related to invasive procedures that are extremely deadky.^4^

Health systems must be prepared to face the risks arising from exposure to health technologies, by using integrated actions to minimize damage caused by intervention with regard to the identified risk factors.^6^ Therefore, the need to assess work processes is identified, considering the potential impact of recent changes in ventilatory practice and patient care when assessing the epidemiology and incidence of AE associated with MV.^7^

Considering the limited number of studies with children and adolescents on the subject, the present study aimed to identify the prevalence and factors associated with AE related to invasive MV in patients admitted to the Pediatric Intensive Care Unit (PICU) of a public tertiary hospital.

## METHOD

This is a cross-sectional study with data collection from July 2016 to June 2018, carried out at the PICU of a tertiary public university hospital, which admits patients from 29 days of life to 18 years of age. Patients submitted to MV in the PICU in the defined time interval were included. Researchers collected data daily during the follow-up from records made in the care routine by the physiotherapy team and other care members. In this study, demographic (sex, age, weight, origin), clinical (primary diagnosis, reasons for admission, deaths) and ventilatory characteristics (cause of MV, type of artificial airway, MV time, presence of cuff, positive inspired pressure (PIP), positive end expiratory pressure (PEEP) and inspired oxygen fraction (IOF_2_)).

In the present study, the following AE were evaluated during follow-up: VAP,^8^ post-extubation stridor, tracheomalaceae, dysphagia, post-extubation aspiration, atelectasis immobility, pneumothorax, unplanned extubation (UPE), decannulation, OTT obstruction, extubation failure, nasal ulceration, sinus and/or ear infections, gastric hemorrhage due to stress, compression injury, selective OTT, exchange of OTT and pressure of cuff above 30 cmH_2_O^9^ and cardiovascular instability secondary to high parameters of MV.

To identify the degree of damage, we used the Classification of the World Health Organization (WHO),^10^ which defines it as mild, moderate, severe and deadly.

1. Mild: Mild symptoms, loss of function or minimal or moderate damage, of rapid duration, and only minimal interventions required.
2. Moderate: Symptomatic patient, requiring intervention.
3. Severe: Symptomatic patient, need for intervention for life support, or large clinical/surgical intervention, causing decreased life expectancy, with great damage or loss of permanent or long-term function.
4. Deadly: Within the odds, in the short term the event caused or accelerated death.

This study was approved by the Research Ethics Committee (CEP), with Report No. 2.093.157.

A statistical analysis was performed using the statistical program Statistical Package for the Social Sciences (SPSS) version 20.0 (IBM Corp., Armonk, New York, United States). Categorical variables were expressed by absolute frequency and percentages. Continuous variables without normal distribution were expressed as medians and interquartile range 25-75% (IQR; 25-75%) and were compared using the nonparametric Mann-Whitney test. The comparison of categorical variables was performed using the asymptotic Pearson’s chi-square test (when 20% of the expected value is between 1 and 5) and the exact Pearson’s chi-square test (when more than 20% of the expected value is between 1 and 5). Probability was considered to be significant when it was less than 0.05 (p <0.05).

The statistical method for multivariate analysis of the factors associated with AE was logistic regression. For the continuous variables, when transformed into categorical variables, the identification of cutoff points (CPs) was performed by an analysis of the Receiver Operating Characteristic Curve (ROC curve). Only the CPS with Area Under the Curve (AUC)>0.50 were investigated. The discriminatory cut-off point was determined by the best relationship between sensitivity and specificity that presented the least amount of discrepancy.

The statistical method for multivariate analysis of the factors associated with AE was logistic regression. All variables with p£0.20 in the univariate analysis were included in the multivariate analysis. In the evaluation of the logistical model, step by step, the variables with the highest p values were removed until all significant variables remaining at the 0.05 level remained in the final model. The risk effect measure used was Odds Ratio (OR), with a 95% confidence interval (95%CI) for the variables associated with the first episode of AE. The quality of fit was assessed using the Hosmer & Lemeshow test.

Regarding the sample size, based on the literature findings that showed AE ranging from 40 to 51.3%,^3^ ^,^ ^11^ ^,^ ^12^ ^,^ ^13^ ^,^ ^14^ ^,^ ^15^ the sample calculation should have had an “n” between 260 and 664 patients. In this study, considering the presence of AE around 20%, with a confidence interval amplitude of 0.10 and a confidence level of 95%, the minimum “n” so that statistical objectivity would not be impaired, was 246 children.

## RESULTS

**Figure 1:**
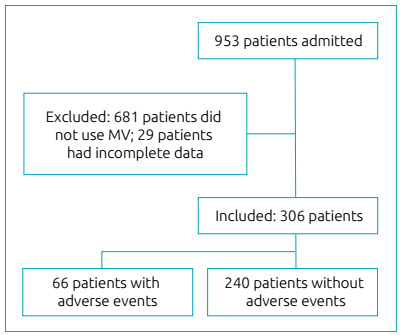
Flowchart of patients admitted to the Pediatric Intensive Care Unit and the sampling process for the cross-sectional study to assess adverse events in the period from July 2016 to June 2018.

During the analyzed period, 953 patients were admitted to the PICU, of which 335 (35.2%), were submitted to invasive MV, and of these, 29 were excluded (29/335 - 8.6%) due to incomplete data during follow-up (Figure 1). Thus, 306 patients were included, with a total ventilation time of 2,155 days. The median age of the group was 24 months (IQR 25-75%: 8-96), with 158 (51.6%) male patients and one with a median weight of 12 kg (IQR 25-75%: 6-23.5). Regarding the reason for admission, 163 (53.3%) patients came in during the postoperative period and 143 (46.7%) came in for clinical causes. Surgical cases were pediatric general surgery in 79 (48.5%) patients; neurosurgery in 32 (19.6%); cardiovascular surgery in 37 (22.7%); orthopedic surgery in eight (4.9%); and ENT surgery in seven (4.3%).

Patients had a median hospitalization of six days (IQR 25-75%: 3-13), Score Pediatric Index Mortality 3 (PIM 3) at admission of 1.7 (IQR 25-75%: 0.87-6.95) and overall mortality of 18.3% (56/306). As for artificial airway, 282 (92.2%) patients used OTT and 24 (7.8%) were given ventilation via cannula tracheostomy (TQT). Of the total of 282 patients with TTO, 175 used TTO with a cuff, of which 34 (19.4%) presented AE, while in the group whose TTO did not have a cuff, 20 had AE (p=0.920).

Among the causes for the use of MV, the main one was postoperative, in 163 (53.3%) patients, followed by respiratory failure, in 60 (19.6%), lower levels of consciousness in 39 (12.7%), hemodynamic instability in 18 (5.9%), post-procedure in 13 (4.3%) and post-cardiorespiratory arrest (PCA) in 13(4.3%). Regarding the ventilatory parameters, the PIP had a median of 16 cmH_2_O (IQR 25-75%: 15-19), the PEEP had a median of 5 cmH_2_O (IQR 25-75%: 5-6), FIO_2_ had a median of 35% (IQR 25-75%: 30-45) and tidal volume (TV) had a median of 8 mL/kg (IQR 25-75%: 7-9). The median total duration of MV was 38 hours (IQR 25-75%: 16-120) (Table 1).

**Table 1:** Demographic and clinical characteristics of patients on mechanical ventilation under study from July 2016 to June 2018.

	Total (n = 306)	Adverse event		p-value
		Present (n = 66)	Absent (n = 240)	
Male [n (%)]	158 (51.6%)	33 (20.9%)	125 (79.1%)	0.764
Weight* (kg)	12 (6-23.5)	10.9 (5-16.5)	12 (7-26)	0.540
Age* (months)	24 (8-96)	16 (5-72)	36 (8.25-96)	0.028^b^
Admission [n (%)]				0.548^a^
Clinic	143 (46.7%)	33 (23.1%)	110 (76. 9%)	
Surgical	163 (53.3%)	33 (20.2%)	130 (79.8%)	
Death [n (%)]	56 (18.3%)	10 (17.9%)	46 (82.1%)	0.455
Score PIM 3*	1.7 (0.87-6.9)	2.1 (0.95-7.2)	1.6 (0.82-6.9)	0.461
Days of hospitalization*	6 (3-13)	8 (4.75-15)	5 (3-11)	0.001
Length of hospital stay ≥7 days	142 (46.4%)	43 (30.3%)	99 (69.7%)	0.001
Sedation time* (hours)	25 (10-95.25)	46.5 (13.25-154)	24 (9.25-83.75)	0.030
MV time* (hours)	38 (16-120)	72 (23.75-216)	36 (15.25-100)	0.008
MV ≥50 hours [n (%)]	134 (43.8%)	39 (29.1%)	95 (70.9%)	0.005
TV* (mL/kg)	8 (7-9)	7 (-8)	8 (7-9)	0.310
PIP * (cmH_2_O)	16 (15-19)	17 (15-19.25)	16 (15-19)	0.388
PEEP * (cmH_2_O)	5 (5-6)	6 (5-6.25)	5 (5-6)	0.084
FiO_2_* (%)	35 (30-45)	37 (39-45)	35 (28-45)	0.122
CA time* (hours)	28 (10.5-96)	59.5 (18.5-170.3)	24 (10-84)	0.002

PIM 3: *Pediatric Index Mortality Score* 3; MV: mechanical ventilation; TV: tidal volume; PIP: positive inspired pressure; PEEP: positive end-expiratory pressure; FiO_2_: fraction of inspired oxygen; CA: controlled assistance; ^a^chi-square test; ^b^Mann-Whitney test; *values expressed as median (interquartile range 25-75%).

The occurrence of AE was observed in 66 (21.6%) patients, of which 11 (16.7%) suffered from two AEs, totaling 77 events (36 AE for one thousand days of ventilation). The prevalent AE was the stridor after extubation, with 20 events (25.9%); followed by UPE, with 13 (16.9%); obstruction of OTT or tracheostomy cannula, with nine (11.7%); Selective OTT, with six (7.8%); and extubation failure, with six (7.8%). The episodes occurred most commonly in the afternoon shift (49.3%) and with a degree of mild damage (54.6%) (Table 2).

**Table 2:** Characteristics of 77 adverse events found in patients on mechanical ventilation from July 2016 to June 2018.

Characteristics of adverse events	n (%)
Adverse events	
Post-extubation stridor	20 (25.9%)
Unplanned extubation	13 (16.9%)
OTT/TQT obstruction	9 (11.7%)
Selective orotracheal tube	6 (7.8%)
Extubation failure	6 (7.8%)
High cuff pressure	5 (6.5%)
Atelectasis	5 (6.5%)
Pneumothorax	5 (6.5%)
OTT/TQT exchange	4 (5.2%)
Pneumonia, associated with mechanical ventilation	3 (3.9%)
Unplanned decannulation	1 (1.3%)
Shift	
Afternoon	35 (49.3%)
Night	20 (28.2%)
Morning	16 (22.5%)
Degree of damage	
Mild	42 (54.6%)
Moderate	17 (22.1%)
Severe	12 (15.6%)
None (incident without damage)	6 (7.8%)
Death	0 (0%)

OTT: orotracheal tube; TQT: tracheostomy.

Comparing the occurrence of AE in the univariate analysis, a statistically significant difference was demonstrated when age (p = 0.028), length of hospital stays (p = 0.001), duration of sedation (p = 0.030), time of MV (p = 0.0085) and time in assist-controlled mode (p = 0.002) were evaluated (Table 1).

In the analysis of the area of the ROC curve, it was observed that the best CP regarding patients who were more likely to suffer an AE, occurred after 50 hours of MV and after seven days of hospitalization. They were continuous variables that presented AUC> 0.5. The length of stay from seven days onwards showed AUC of 0.63 (95%CI 0.56-0.70), with a sensitivity of 65.2%, a specificity of 58.7%, a positive predictive value of 1.57, and a negative predictive value of 0.59. Time above 50 hours of MV showed an AUC of 0.60 (95%CI 0.53-0.69), a sensitivity of 57.6%, a specificity of 60.4%, a positive predictive value of 1.45, and a negative predictive value of 0.70.

The final multivariate logistic regression showed that children hospitalized for seven or more days were 2.63 times more likely to suffer from AE (p = 0.001) (Table 3). The result of the Hosmer-Lemeshow test statistic showed p=0.887.

**Table 3:** Multivariate logistic regression analysis of the presence of adverse events.

Variables	Odds Ratio	95%CI	p-value
Length of hospital stay ≥7 days	2.63	1.49-4.66	0.001
MV time> 50 hours	1.02	0.98-1.04	0.127
Sedation time (hours)	0.99	0.88-1.02	0.186
PEEP (cmH_2_O)	1.03	0.87-1.23	0.687
FiO_2_ (%)	0.98	0.96-1.08	0.371
Assist-controlled time (hours)	0.97	0.95-1.01	0.112
Age (months)	0.79	0.59-1.04	0.100

95%CI: 95% confidence interval; MV: mechanical ventilation; PEEP: positive end-expiratory pressure; FiO_2_: fraction of inspired oxygen.

## DISCUSSION

The MV is an invasive measure applied in urgent situations such as life support, and it can cause complications and AE. The type and number of AEs and complications depends on the characteristics of the patients, the experience of the team, and their resources at each center.^11^ Studies in the pediatric age range are still restricted, with a limited number of cases describing VAP more commonly, reinforcing the purpose and relevance of this study to assess the prevalence, types of events, and factors associated with the occurrence of AE related to MV in children and adolescents.^3^ ^,^ ^11^ ^,^ ^12^ ^,^ ^13^ ^,^ ^14^ ^,^ ^15^ ^,^ ^16^ ^,^ ^17^ The results of the present study showed a significant number of AEs that can be prevented, identifying the length of hospital stay as a risk factor to be monitored and evaluated regarding the prevention of these occurrences.

In the present study, a prevalence of 21.6% of AE was observed (36 AE per thousand days of MV), which is lower than the studies by Kendirli et al.^12^ (42.8%), Meligy et al.^13^ (39.9% or 29.5 AE per thousand days of MV), De Jesus et al.^3^ (51.3%) and Principi et al. (40% or 114 AE per thousand days of MV).^15^ We also found that 16.6% of patients suffered two AE, similar to the study by Meligy et al.,^13^ in which 11.9% suffered more than one AE. The variation in prevalence found can be influenced by the selection made by each author regarding which events to analyze. Thus, the parameters associated with the culture of safety and surveillance of events as a quality indicator also vary greatly between services.

The most common AE found in this study was stridor after extubation (25.9% of events), similar to the studies by Dave et al.,^16^ who also found stridor as the predominant event in 15.7% of patients. Principi et al.^15^ and Anitha et al.^17^ also showed similar values, with 13.3 and 15.8%, respectively. The main factors that lead to stridor were prolonged MV time, trauma related to intubation and younger ages, especially children below the age of four years old.^18^ ^,^ ^19^ ^,^ ^20^ Jansaithong^21^ also mentions the size of the OTT, episodes of coughing, excessive head movements and infections of the airways. Studies have shown no association between tubes with and without cuff and stridor after extubation.^18^ ^,^ ^22^ ^,^ ^23^

Post-extubation upper airway obstruction is a frequent complication in the pediatric population, and is estimated to be responsible for one third of extubation failures.^24^ As a preventive measure for this AE, there are airway permeability tests,^25^ but there is still no evidence in the literature to be applied routinely in the pediatric age group, which is why we have not used it in our service routine.

UPE occurred in 13 (16.9%) patients, and there is a wide range of frequency among the various studies in the literature. In the study by Jesus et al.,^14^ UPE was the most frequent event, with 31.9%, while Principi et al.^15^ and Dave et al.^16^ found, respectively, 3.3 and 3.4%. According to Da Silva et al.,^26^ the risk factors for UPE can be related to the patient or the process and the unit. The factors related to the patient include the level of consciousness (restlessness, agitation, use of physical restraints). The risk factors related to the process, on the other hand, include activities that involve the carefulness of the patient care team, such as procedures, manipulations of the critically ill patient and care with the fixation of OTT.^27^ On the other hand, the risk factors related to the unit are associated with the number of nurses responsible for the patient, the workload and nursing assignment overload.^26^

The obstruction of OTT/tracheostomy (TQT) was observed in nine (2.9%) patients, a small index compared to the data by Dave et atl.^16^ (6.5%) or Arriagada et al.^11^ (11.3%). Despite its low frequency, extreme importance should be given to preventing this event, as it leads to other AEs, which is the exchange of OTT/TQT without prior planning. This urgent procedure can lead to hypoxemia and cardiorespiratory arrest. In addition, obstruction of OTT/TQT by a thick secretion stopper is almost always an avoidable event. It can be avoided by checking the humidification in the mechanical ventilators and through more rigorous observation with patients with thicker secretion or bleeding through OTT.

In this study, length of stay was identified as a risk factor for AE, which was also observed in the studies by Anitha et al.,^17^ Torres-Castro,^28^ De Jesus et al.,^14^ Meligy et al.^13^ and Ramirez.^29^ Therefore, the importance of managing these patients for the shortest possible hospital stay remains evident. However, this finding should be evaluated with caution in this study, since the association of longer hospital stay with a higher risk of AE may not be a variable that is independent of the severity of the clinical condition or the reason for hospitalization in the PICU, since in the adjustment of the final model, these variables were not included. As such, it is a limitation to be considered.

It is often reported in the neonatal population that the younger the age, the greater the chances of suffering an AE related to MV.^30^ Although age did not enter the final multivariate model of this study, this variable had a statistically significant difference in the univariate analysis. This may be associated with the fact that younger children are more difficult when dealing with sedation, they use artificial airways that are smaller in caliber, they salivate more, in addition to specific anatomical characteristics of the airways that make them more prone to events.^30^ ^,^ ^31^

AE is characterized by unintentional injury or damage caused to the patient from the assistance intervention and not by the underlying disease, while complications can originate from the underlying disease. However, the relevant similarity is that AE and complications associated with the diseases can be avoided by implementing prevention protocols. In the literature, the protocols for preventive measures for AE related to MV in general are more focused on specific events, such as VAP^8^ and UPE.^32^ Establishing prevention protocols is important, since the occurrence of AE, especially VAP, post-extubation stridor and UPE, results in an increase in the length of hospital stay, which increases the chance of new events occurring, especially considering that, in this study, an association was found between the occurrence of events and longer hospital stay.

This study, because it was carried out only one center, presents a limitation regarding the number of patients included, which may influence the results, especially for the multivariate analysis. This limitation was also found in studies on the subject in the literature.^3^ ^,^ ^11^ ^,^ ^12^ ^,^ ^13^ ^,^ ^14^ ^,^ ^15^ ^,^ ^16^ ^,^ ^17^ Despite the importance of studying AE related to MV, the number and type of AE in a single center can limit extrapolations, reinforcing the need for multicenter studies that elucidate the main risk factors and enable preventive measures.

It was concluded, in this study, that children are more likely to suffer an AE when exposed to a hospitalization time equal to or greater than seven days, which suggests greater attention to this population and the need to implement protocols with greater rigidity regarding care related to MV. Among the AE found, the post-extubation stridor and the UPE were the main ones, and both were perfectly capable of being prevented.
